# Fast approximate hierarchical clustering using similarity heuristics

**DOI:** 10.1186/1756-0381-1-9

**Published:** 2008-09-22

**Authors:** Meelis Kull, Jaak Vilo

**Affiliations:** 1 Institute of Computer Science, University of Tartu, Liivi 2, 50409 Tartu, Estonia; 2 Quretec Ltd. Ülikooli 6a, 51003 Tartu, Estonia

## Abstract

**Background:**

Agglomerative hierarchical clustering (AHC) is a common unsupervised data analysis technique used in several biological applications. Standard AHC methods require that all pairwise distances between data objects must be known. With ever-increasing data sizes this quadratic complexity poses problems that cannot be overcome by simply waiting for faster computers.

**Results:**

We propose an approximate AHC algorithm HappieClust which can output a biologically meaningful clustering of a large dataset more than an order of magnitude faster than full AHC algorithms. The key to the algorithm is to limit the number of calculated pairwise distances to a carefully chosen subset of all possible distances. We choose distances using a similarity heuristic based on a small set of pivot objects. The heuristic efficiently finds pairs of similar objects and these help to mimic the greedy choices of full AHC. Quality of approximate AHC as compared to full AHC is studied with three measures. The first measure evaluates the global quality of the achieved clustering, while the second compares biological relevance using enrichment of biological functions in every subtree of the clusterings. The third measure studies how well the contents of subtrees are conserved between the clusterings.

**Conclusion:**

The HappieClust algorithm is well suited for large-scale gene expression visualization and analysis both on personal computers as well as public online web applications. The software is available from the URL

## Background

Various types of biological data resulting from high-throughput experiments require analysis, often consisting of many steps. The first steps tend to be unsupervised and require little or no input from the user, while the further steps need more human-computer interaction. One possible starting point of interaction is showing an overview of the data to the user, frequently achieved using clustering.

Partitioning-based clustering methods like K-means split the data into non-overlapping clusters [1]. In this article, we concentrate on hierarchical methods that model the data in a tree structure and thus leave more freedom to the user.

Probably the most well-known hierarchical clustering method is agglomerative hierarchical clustering (AHC). To begin with, AHC treats each data object as a separate cluster. The following agglomeration steps iteratively merge the two nearest clusters. Simultaneously, the clustering tree (*dendrogram*) is built from leaves towards root, where merging of clusters is depicted as a common parent for two subtrees. Finally, the user is shown the dendrogram, possibly together with data objects visualized at each leaf. There are different versions of AHC, depending on how the distance between clusters is measured. It can be defined as the distance between the closest or furthest objects, resulting in single or complete linkage AHC, respectively. Other known strategies include UPGMA (unweighted pair-group method using arithmetic averages) and WPGMA (weighted pair-group method using arithmetic averages) clustering. For a more detailed exposition refer to Legendre & Legendre [2].

AHC is often used to visualize microarray gene expression data. Originally suggested by Eisen *et al. *[3], there now exist many tools for performing AHC of gene expression data, *e.g. *Cluster 3.0 [4] coupled with Java Treeview [5], MultiExperiment Viewer (MeV 4.0) [6], and EP:NG [7] with clustering tools from EPCLUST 1.0 [8]. An important issue of using this simple and intuitive procedure is its speed. Some of the best implementations can achieve quadratic speed in the number of data objects [9]. At least quadratic running time is required for general distance measures, as exemplified by the need of first finding a single closest pair of objects from all possible candidate pairs.

As will be shown in the Results section, full AHC of genes in medium-sized expression datasets can be performed in a couple of minutes on a workstation computer. Clustering large datasets with 20000+ genes and several thousands of conditions can already take several hours. One possible workaround involves decreasing the size of the dataset by filtering out the genes that change too little over the conditions. However, dropping a large set of data in a very early stage of study might be undesirable. Secondly, when the number of experimental conditions is large, almost all genes show some differential expression. Another possibility is to first run K-means and then apply AHC on the centers of obtained clusters. The downside is that K-means is inherently limited in the choice of the distance measure. K-medoids is free from this constraint, but has quadratic complexity [10].

To our knowledge, significant speedups to AHC have only been achieved in specialized computational environments, such as parallel computing [11] and graphics hardware acceleration [12]. Another direction of research has involved the development of hierarchical clustering methods in a different framework, such as DIANA [10] or SOTA [13]. Both of these are divisive hierarchical clustering algorithms as opposed to AHC. There is a clear need for fast AHC for interactive tools, web services and other applications, running on personal and workstation computers or servers with many simultaneous users.

The speed problem is caused by the fact that all pairwise comparisons have to be performed between the data objects. Each comparison involves a distance calculation using the distance measure specified by the user, where short distances correspond to more similar objects. Not a single pair can be omitted, because already in the first iteration AHC uses each of the pairwise distances to find the closest pair. In the following we take the only route to a significant speedup and drop the requirement of calculating all pairwise distances. This certainly affects the dendrogram and the conclusions that can be drawn from it. In this article, we present a fast approximate hierarchical clustering algorithm which uses similarity heuristics to retain high quality of clustering.

Our approximate hierarchical clustering algorithm first calculates a subset of pairwise distances and then performs agglomerative clustering based on these distances. The logic of the agglomeration part is straightforward – make merging decisions based on the known distances only. The quality of the resulting clustering depends heavily on the subset of distances chosen in the first step. Therefore, we use heuristics to rapidly find pairs of similar data objects. The heuristics are based on the observation that if two objects are close enough to each other then the distance to any third object from both of these is approximately the same. We turn this observation upside down and look for pairs of objects which are approximately at the same distance from several other objects (which we refer to as *pivots*). These pairs are more probably similar and form the subset of pairs for which the distance is calculated. Experiments show that adding a random set of pairs to the pool further raises the quality of clustering. Pivots have earlier been used in the database community for several similarity search algorithms [14].

The running time of the HappieClust algorithm can be easily controlled by choosing the number of distances that are to be calculated and used in the clustering. Such a feature is very useful in web-based applications where users expect fast response time.

The experiments to evaluate speed and quality of HappieClust have been carried out using two datasets: DNA microarray survey of gene expression in normal human tissues [15] and human gene expression atlas of public microarray data [16] (accession E-TABM-185 of ArrayExpress [17]).

## Methods

### Approximate hierarchical clustering

Suppose we have a dataset *X *with *n *data objects, *X *= (*x*_1_, *x*_2_,...,*x*_*n*_) and a user-defined distance measure *d*(*x*_*i*_, *x*_*j*_) to state how similar the two objects *x*_*i *_and *x*_*j *_are, for any *i *and *j*. We assume the distance measure to be a semi-metric, *i.e. *it must be non-negative and symmetric and the distance from an object to itself must be 0:

$$\begin{array}{ll} d(x_{i},x_{j})\geq 0 & i,j=1,\ldots ,n;\\ d(x_{i},x_{j})=d(x_{j},x_{i}) & i,j=1,\ldots ,n;\\ d(x_{i},x_{i})=0 & i=1,\ldots ,n. \end{array}$$

The dataset can have duplicate objects and we do not require the triangle inequality *d*(*x*_*i*_, *x*_*j*_) ≤ *d*(*x*_*i*_, *x*_*k*_) + *d*(*x*_*k*_, *x*_*j*_).

Standard agglomerative hierarchical clustering starts off clustering this data by putting each of the data objects *x*_*i *_in a singleton cluster *C*_{*i*} _= {*x*_*i*_} and then keeps on joining the closest pair of clusters *C*_{*i*} _∪ *C*_{*j*} _= *C*_{*i*, *j*} _until there is only one large cluster *C*_{1,2,...,*n*}_. The distance between clusters $C=C_{\left\{i_{1},i_{2},\ldots ,i_{r}\right\}}$ and $C^{\prime }=C_{\left\{j_{1},j_{2},\ldots ,j_{s}\right\}}$ can be measured in several ways. Three popular methods are single, complete and average linkage:

$$\begin{array}{ll} d_{\min}(C,C^{\prime })=\underset{x\in C,y\in C^{\prime }}{\min}d(x,y) & \left(\text{single}\right)\\ d_{\max}(C,C^{\prime })=\underset{x\in C,y\in C^{\prime }}{\max}d(x,y) & \left(\text{complete}\right)\\ d_{\text{ave}}(C,C^{\prime })=\frac{1}{\left|C|\cdot |C^{\prime }\right|}\sum_{x\in C,y\in C^{\prime }}d(x,y) & \text{(average),} \end{array}$$

where |*C*| denotes the number of objects in cluster *C*. It is seen that the distance between clusters is the minimum (or maximum or average) of the distances between one object from the first and another from the second cluster.

It is important to note that the distances to a newly merged cluster *C' *∪ *C" *can be easily calculated using distances to the clusters *C' *and *C"*:

$$\begin{array}{lll} d_{\min}(C,C^{\prime }\cup C^{\prime \prime }) & = & \min(d_{\min}(C,C^{\prime }),d_{\min}(C,C^{\prime \prime }))\\ d_{\max}(C,C^{\prime }\cup C^{\prime \prime }) & = & \max(d_{\max}(C,C^{\prime }),d_{\max}(C,C^{\prime \prime }))\\ d_{\text{ave}}(C,C^{\prime }\cup C^{\prime \prime }) & = & \frac{\left|C^{\prime }|\cdot d_{\text{ave}}(C,C^{\prime })+|C^{\prime \prime }|\cdot d_{\text{ave}}(C,C^{\prime \prime }\right)}{\left|C^{\prime }\cup C^{\prime \prime }\right|}. \end{array}$$

Standard agglomerative hierarchical clustering algorithm can be translated into the language of graphs by representing clusters as nodes and distances between them as edges. When put this way, hierarchical clustering starts off with a complete graph with all the possible pairwise distances, looks for the shortest edge (pair of most similar clusters), and joins the end nodes (clusters) of this edge. While doing so, the distances to other nodes are recalculated using the formulas given above. The algorithm keeps on finding the shortest edge and joining respective nodes until there is only one node left.

Our approximate hierarchical clustering works exactly the same way except that the graph is not required to be complete, *i.e. *the algorithm works even if only a subset of pairwise distances is known. This is a clear advantage in case of large datasets and/or complex distance measures. Therefore, we redefine the distance between clusters to be the minimum (or maximum or average) of only the known distances measured between the objects of the two clusters. If no distances are known at some point in the algorithm, *i.e. *the distance graph is not connected, two randomly chosen clusters are joined. This situation can be avoided by calculating enough distances because almost all graphs with *n *nodes and at least *n *log *n *edges are connected [18]. When all distances are known, approximate hierarchical clustering coincides with the standard hierarchical clustering.

We have developed a simple algorithm (see Algorithm 1) for performing approximate hierarchical clustering. Step 1 of this algorithm takes O(*m*) time, steps 3, 8, 9 take O(log *m*) time [19], and steps 4, 6, 12 take O(1) time, where *m *is the number of known distances. As steps 8 and 9 are repeated at most *m *times, the overall time complexity is O(*m *log *m*). The largest data structure which needs to be stored by the algorithm is the heap *H*, so the required memory is O(*m*).

**Algorithm 1 **Approximate hierarchical clustering using a given subset of pairwise distances

**Require: ***m *distances between *n *data objects (a graph with *n *nodes and *m *edges), linkage method.

1:   build a heap *H *of all edges

2:   **while ***H *is not empty **do**

3:      (*u*, *v*) ⇐ *H *{take the shortest edge from the heap}

4:      swap *u *and *v *if *u *has more outgoing edges than *v*

5:      **for all **edges (*u*, *x*) going out from *u ***do**

6:         change (*u*, *x*) into (*v*, *x*), keep the same length

7:         **if **(*v*, *x*) is a duplicate edge **then**

8:            calculate new length of (*v*, *x*) from old lengths

9:            remove the duplicate from graph and heap

10:         **end if**

11:      **end for**

12:      remove node *u*

13:   **end while**

### Similarity heuristics

Not all distances are of equal importance to the result of hierarchical clustering. Many distances can be omitted from the distance graph while the dendrogram structure will remain exactly the same. But, for example, removing the shortest distance will unavoidably lead to a different choice at the first join and thus to a different dendrogram. This gives a clear hint that if we cannot afford to calculate all pairwise distances, the distances we choose should be biased towards shorter distances. At the same time, some long distances should be known, in order to have the "big picture" of data. For example, in case of several large clusters we still need to know which of these are closer to each other, in order to get the dendrogram right. In the following, we calculate some of the distances randomly and some using the similarity heuristics which we describe next.

**Figure 1:**
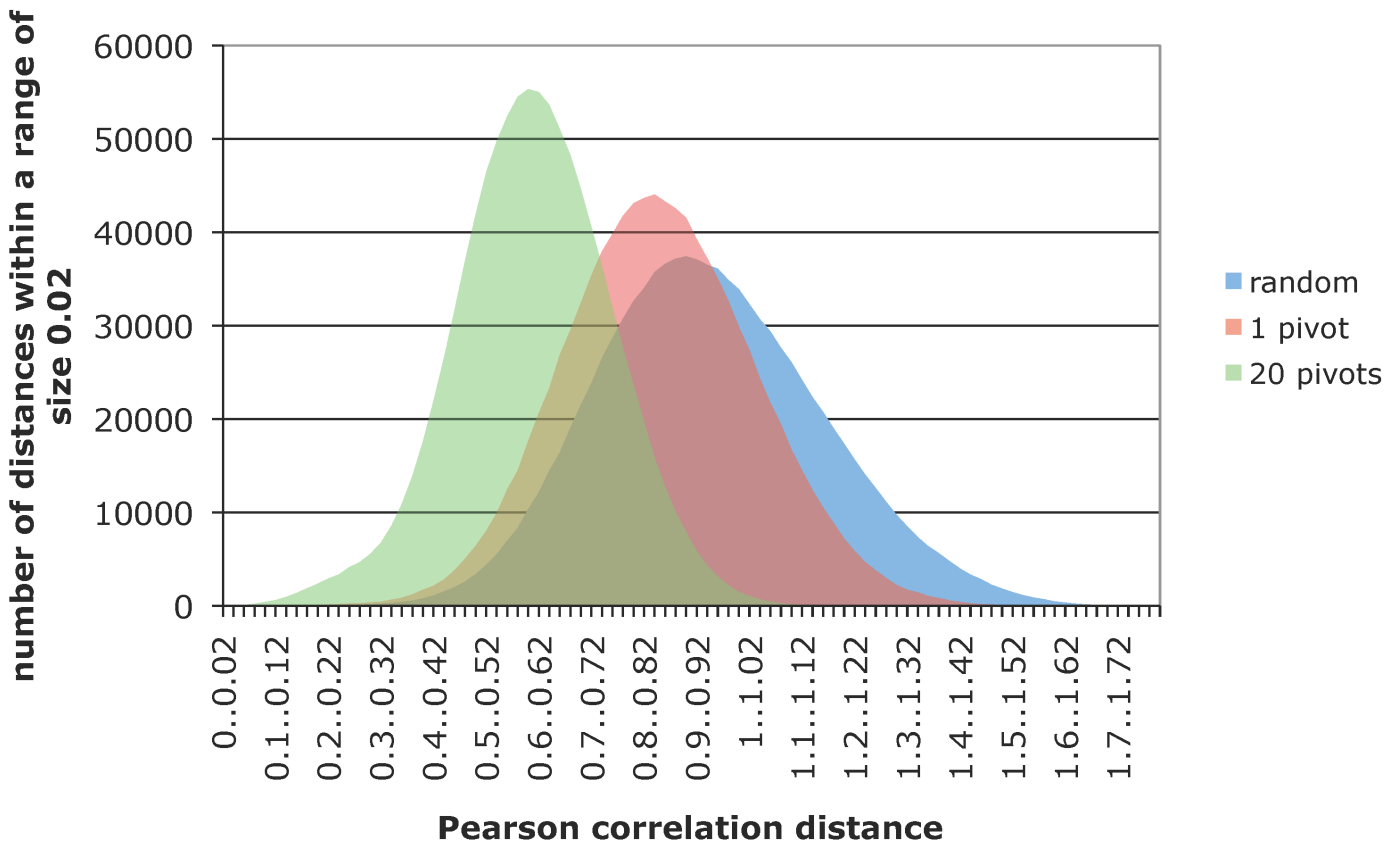
**Effect of similarity heuristics**. Distribution of 1 million pairwise Pearson correlation distances in the Shyamsundar05 dataset (described in the Results) chosen randomly *vs. *obtained with similarity heuristics using 1 or 20 pivots.

The main idea of our heuristics is the following. Suppose that two objects are similar to each other, *d*(*x*_*i*_, *x*_*j*_) <*δ*. It is now quite natural to assume that any third object *x*_*k *_is of similar distances to *x*_*i*_, *x*_*j*_, *e.g. *|*d*(*x*_*k*_, *x*_*i*_) - *d*(*x*_*k*_, *x*_*j*_)| <*ε*. Note that due to the triangle inequality, the above holds for metric distance measures with *ε *= *δ*. Let us now fix *x*_*k*_, which we call a *pivot*, and look at all the pairs (*x*_*i*_, *x*_*j*_) for which |*d*(*x*_*k*_, *x*_*i*_) - *d*(*x*_*k*_, *x*_*j*_)| <*ε*. In Figure 1 (1 pivot) we have chosen *ε *so that there are 1 million pairs with |*d*(*x*_*k*_, *x*_*i*_) - *d*(*x*_*k*_, *x*_*j*_)| <*ε*. The smoothened histogram of these distances is compared with a random sample of 1 million pairwise distances, illustrating the basic property required for our heuristics – pairs that are of similar distances to the pivot are biased towards shorter distances.

In our similarity heuristics we first choose *q *pivots *p*_1_, *p*_2_,...,*p*_*q *_randomly from the dataset *X*. Next we calculate all the *n *× *q *distances *d*_*ik *_= *d*(*x*_*i*_, *p*_*k*_) from each of the data objects to each of the pivots. We then seek object pairs (*x*_*i*_, *x*_*j*_) which are of similar distances from each of the pivots, *i.e. *|*d*(*p*_*k*_, *x*_*i*_) - *d*(*p*_*k*_, *x*_*j*_)| <*ε *for each pivot *p*_*k*_. Figure 1 confirms our hypothesis that taking more pivots introduces stronger bias towards shorter distances. The conditions |*d*(*p*_*k*_, *x*_*i*_) - *d*(*p*_*k*_, *x*_*j*_)| <*ε *can be rewritten as

$$\overset{q}{\underset{k=1}{\max}}|d_{ik}-d_{jk}|<\varepsilon .$$

On the left side of the inequality we can recognize the Chebyshev distance between *q*-dimensional vectors *d*_*i *_= (*d*_*i*1_,...,*d*_*iq*_) and *d*_*j *_= (*d*_*j*1_,...,*d*_*jq*_). We denote this distance by *c*(*d*_*i*_, *d*_*j*_). Thus we can rewrite the inequality as *c*(*d*_*i*_, *d*_*j*_) <*ε*. To avoid confusion, we refer to *d*(*x*_*i*_, *x*_*j*_) as the distance and *c*(*d*_*i*_, *d*_*j*_) as the *pseudo-distance *between objects *x*_*i *_and *x*_*j*_. Our similarity heuristic requires all pairs (*x*_*i*_, *x*_*j*_) for which the pseudo-distance is less than *ε*. These pairs can be fetched using a similarity join, for which we use the algorithm Epsilon Grid Order (EGO) [20]. EGO splits the *q*-dimensional space into hypercubes of side-length *ε *– any two objects in the same hypercube are similar, any two objects more than one hypercube apart are dissimilar, and objects in neighbouring hypercubes can be either. The hypercubes are sorted in a specific manner called *epsilon grid order *in order to prune the search tree.

### HappieClust

We now have the required notions to describe our approximate hierarchical clustering algorithm HappieClust (Algorithm 2). It requires 5 input parameters: distance measure; joining linkage method (single, average or complete); number of pivots, 0 ≤ *q*; number of distances to calculate, 0 ≤ *m *≤ (*n *- 1)*n*/2; and the proportion of similarity heuristics based distances, 0 ≤ *s *≤ 1. The proportion *s *is achieved by HappieClust only approximately, using the estimation of *ε *in step 1d of Algorithm 2.

**Algorithm 2 **HappieClust – approximate hierarchical clustering using similarity heuristics

**Require: **dataset (size *n*), distance measure, linkage method, number of pivots (*q*), proportion of similarity heuristics based distances (*s*), number of distances to calculate (*m*).

1. **Initialization:**

(a) choose *q *pivots *p*_1_, *p*_2_,...,*p*_*q *_randomly among the data objects

(b) calculate distances from every data object to all pivots

(c) calculate the pseudo-distances between *n *randomly sampled pairs of objects

(d) estimate *ε *based on the pseudo-distances in the sample such that approximately *s*·*m *out of all (*n *- 1)*n*/2 pairwise pseudo-distances would be less than *ε*

2. **Distances based on similarity heuristics:**

(a) find all pairs with pseudo-distance less than *ε *(using EGO)

(b) calculate actual distances between pairs from the previous step

3. **Random distances:**

(a) calculate about (1 - *s*)·*m *additional distances between random pairs of objects to achieve *m *distances in total

4. **Approximate hierarchical clustering:**

(a) run approximate hierarchical clustering (Algorithm 1) on distances from steps 2 and 3

The two first parameters are shared with full hierarchical clustering. As shown in the Results section, values *q *= 20 and *s *= 0.5 seem to work well in all conditions and do not have to be ever changed. Hence, HappieClust has only one additional parameter compared to full hierarchical clustering. This parameter *m *is required to specify the level of approximation and can be estimated from the given time constraints. HappieClust gives the best quality approximation it is able to reach within the given time frame.

## Results

The experimental work of this study is divided into three parts. First, we examine the running time of three full hierarchical clustering tools and HappieClust. Next, we show how the quality of approximation depends on the parameters of HappieClust. Finally, we give results of applying HappieClust on a large gene expression dataset.

As a medium-sized dataset we have chosen a DNA microarray survey of gene expression in normal human tissues [15], hereafter referred to as data=Shyamsundar05. It involves expression values for 15521 genes in 123 conditions. The large dataset is from a study to build a human gene expression atlas of public microarray data [16], data=Lukk08, with 22283 probes in 5896 conditions (accession E-TABM-185 of ArrayExpress [17]). In both cases we have chosen to use average linkage hierarchical clustering with Pearson correlation distance, a common choice for microarray gene expression data.

### Running time experiments

Running time experiments were performed on a laptop computer (MacBook with 2.0 GHz Intel Core Duo processor and 2 GB RAM) and a workstation (64-bit Linux computer with four 2.0 GHz AMD dual core processors and 32 GB RAM). Note that our algorithm has not been parallelised to use multiple processors. We measured separately the time spent on the distance matrix calculation and the time spent on agglomeration. Each measurement was repeated three times and the median was reported.

**Figure 2:**
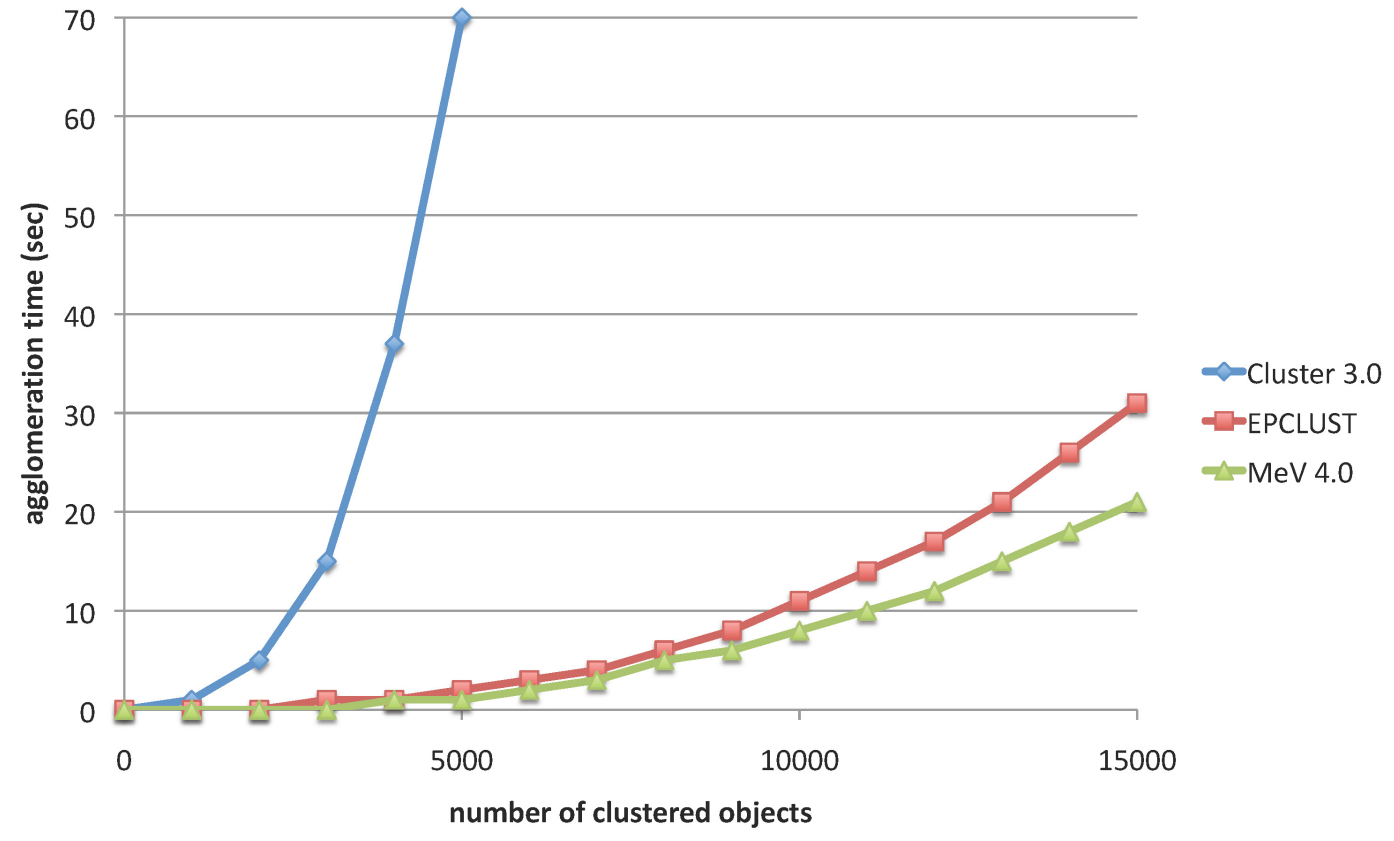
**Speed of hierarchical clustering tools**. Agglomeration times of three hierarchical clustering tools on the laptop computer.

First, we measured the speed of tools Cluster 3.0 [4], MeV 4.0 [6], and EPCLUST 1.0 [8] in order to later compare HappieClust with the fastest tool. The agglomeration times on the laptop with *n *= 1000, 2000,...,15000 randomly generated vectors are plotted in Figure 2 and show that MeV 4.0 is slightly faster than EPCLUST 1.0 while Cluster 3.0 is much slower. Calculation of 100 million Pearson correlation distances between vectors of length *l *took about 5·*l *seconds with MeV, 1·*l *seconds with Cluster 3.0, and 0.5·*l *seconds with EPCLUST 1.0. Summing up the times of distance calculations and agglomeration we see that EPCLUST 1.0 is the fastest tool. For example, hierarchical clustering of 15000 vectors of length 10 takes about 30 + 0.5·10 = 35 seconds with EPCLUST 1.0 and about 20 + 5·10 = 70 seconds with MeV 4.0.

**Figure 3:**
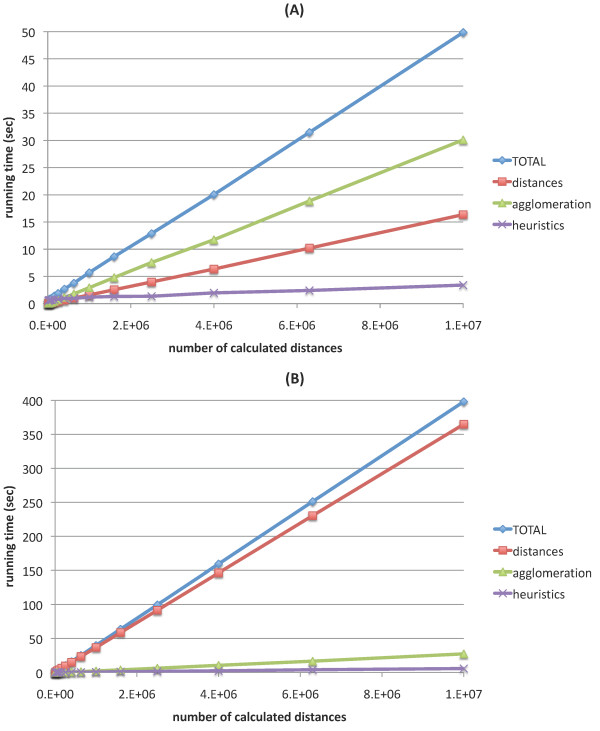
**Speed of HappieClust**. HappieClust running times for *q *= 20, *s *= 0.5 on the workstation, (A) data=Shyamsundar05; (B) data=Lukk08. The running times for full hierarchical clustering using EPCLUST 1.0 are 97 + 48 = 145 seconds and 9580 + 115 = 9695 seconds, respectively.

Next, the running time of HappieClust using different parameters with data=Shyamsundar05 and data=Lukk08 was measured on the workstation computer. As a comparison, we ran EPCLUST 1.0, which clustered data=Shyamsundar05 in 97 + 48 = 145 seconds and data=Lukk08 in 9580 + 115 = 9695 seconds, where the two addends stand for distance calculation time and agglomeration time, respectively. With HappieClust we experimented with *q *∈ {5, 10, 20} pivots and *s *∈ {0, 0.5, 1} proportion of similarity heuristics based distances. For each of the combinations we ran a series of time tests for different numbers of calculated distances, out of all approximately 1.2·10^8 ^and 2.5·10^8 ^pairwise distances in the two datasets. Figure 3 shows the results for *q *= 20 and *s *= 0.5. The running times for other combinations of parameters deviated from these by less than 3 seconds or by less than 30 percent. Our experiments show that the EGO algorithm used for heuristics is fast enough, spending less than 10 percent of the total time. In case of data=Lukk08, most of the time was spent on distance calculations, as the number of conditions in this dataset is large (5896). Agglomeration was the slowest part for data=Shyamsundar05, as this dataset has less conditions (123).

We also tested HappieClust on the laptop. The running times for data=Shyamsundar05 with the above given parameter combinations deviated from the ones on the workstation by less than 3 seconds or by less than 30 percent. EPCLUST 1.0 clustered this dataset in 81 + 37 = 118 seconds, which is 20 percent faster than on the workstation. As data=Lukk08 did not fit in the main memory of the laptop, times for this dataset were not compared.

In conclusion, the experiments have shown that the running times of HappieClust (as well as full AHC) depend almost linearly on the number of calculated distances and very little on other factors. In order to evaluate the quality of approximate clustering, we use two different approaches. The first is a mathematical measure, while the second evaluates whether we lose any important biological information if we cluster gene expression data approximately. The third measure shows how large overlap we can expect from the subtrees of approximate and full AHC dendrograms.

### Quality estimation by joining distance ratio

**Figure 4:**
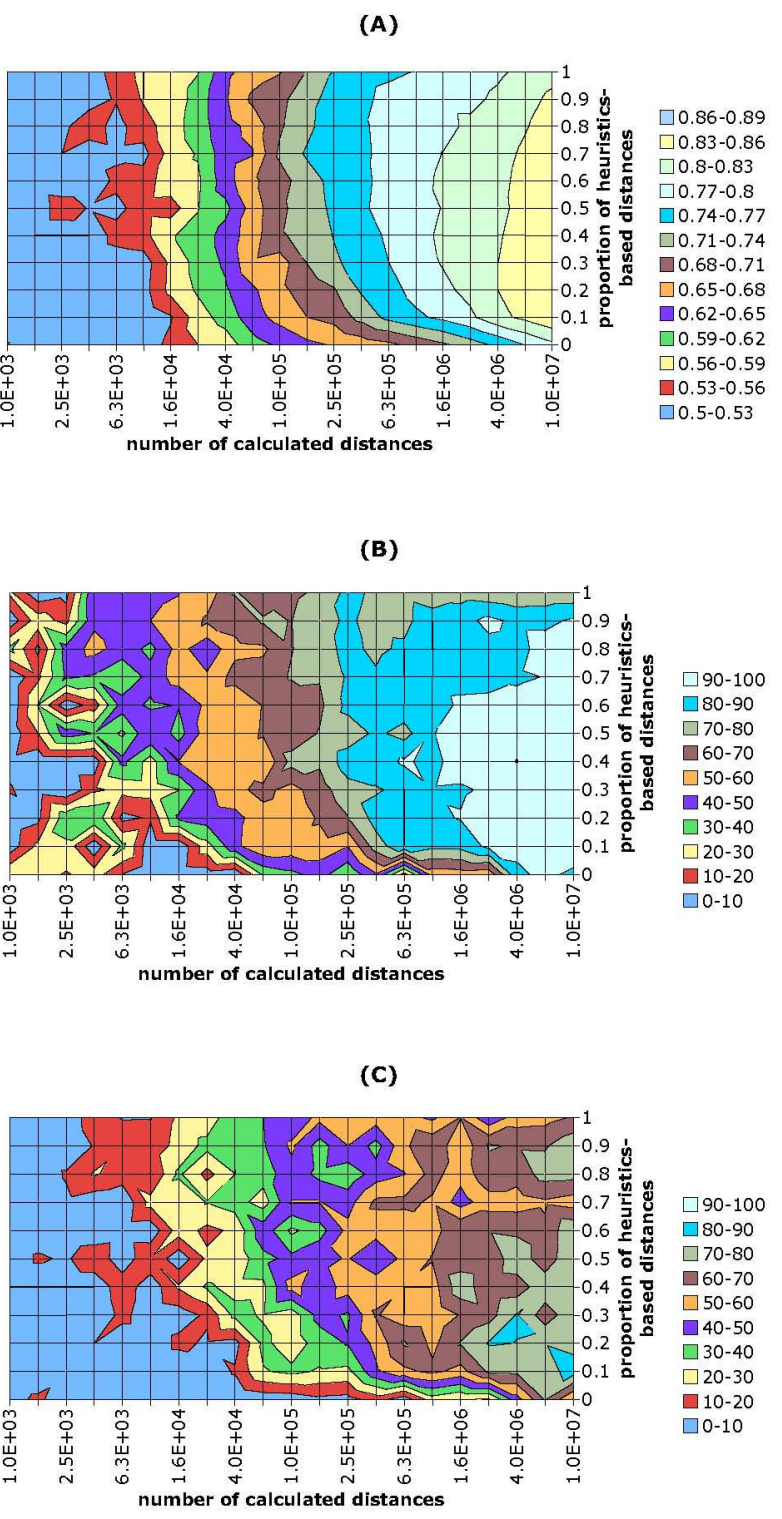
**The influence of parameters on approximation quality with *q *= 20 pivots**. (A) JDR, (B) GO50 and (C) GO25 quality of HappieClust for data=Shyamsundar05 and *q *= 20 pivots.

**Figure 5:**
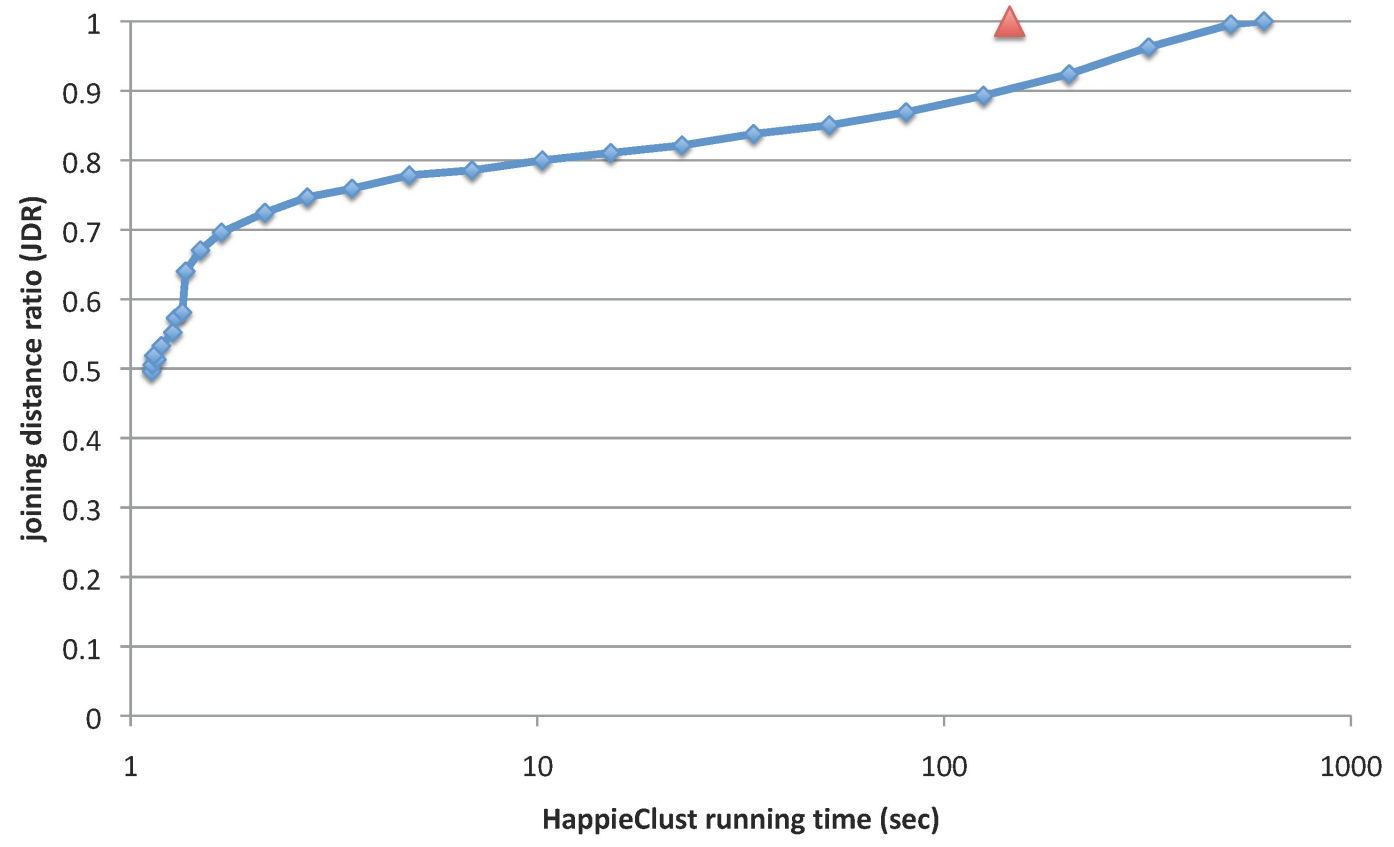
**JDR quality for different running times of HappieClust**. HappieClust JDR quality and running time for data=Shyamsundar05, *s *= 0.5, *q *= 20. HappieClust running times for 10^6 ^and 10^7 ^distances are 6 and 50 seconds, respectively. The time for full AHC is 145 seconds with EPCLUST 1.0 (marked with a triangle) and 612 seconds with HappieClust. This 4-fold difference is due to the overhead in HappieClust used for working with incomplete information.

Full AHC can be viewed as having the objective of minimizing the sum of joining distances. By this we mean the sum where for each agglomeration step we take the distance between the clusters that are merged. Full AHC attempts to minimize this sum by greedily choosing the nearest clusters at each step. In the particular case of single linkage clustering, a minimal spanning tree is constructed that provably minimizes this sum [21]. The difference of approximate AHC is that it holds only partial information. Therefore, the algorithm sometimes joins two clusters for which the true distance (not the estimation by approximate clustering) is larger than for some other pair of clusters. To measure the quality of a dendrogram, we use full AHC as a reference, and divide the sum of joining distances for full AHC by the sum of joining distances in the dendrogram under study. We will refer to this as JDR (joining distance ratio). Quality of the full AHC dendrogram is 1 by definition and smaller values indicate lower quality. Our task with HappieClust is to obtain a dendrogram with quality close to 1 significantly faster than the running time of full AHC. Figure 4A reflects the quality of HappieClust on dataset Shyamsundar05 for *q *= 20 pivots (see Additional file 1 for different numbers of pivots). We have varied the proportion of similarity heuristics based distances, *s *∈ {0, 0.1, 0.2,...,0.9, 1}, and the number of calculated distances. The colors stand for regions of different JDR quality, as indicated on the legend. The figure clearly shows that the more distances are calculated, the higher is the quality of the result, as the colors change from lower to higher values when moving from left to right. Usage of heuristics based distances is definitely justified because for a fixed number of distances, using no heuristics results in the poorest quality. The number of pivots *q *= 20 outperforms *q *= 5 and *q *= 10 [see Additional file 1], reaching the same JDR quality with smaller number of calculated distances. For *q *= 20 (Figure 4A), the optimal proportion of heuristics based distances varies from *s *= 0.1 to *s *= 0.9 for different numbers of calculated distances. However, the quality for *s *= 0.5 is never far from the optimum. We will use *q *= 20 and *s *= 0.5 hereafter as the default choice for these parameters. Larger numbers of pivots will be tested in the subsection about subtree content conservation. Figure 5 shows that for data=Shyamsundar05, high JDR quality of 0.8 was achieved more than an order of magnitude faster than the time of full hierarchical clustering. This required the calculation of about a million distances and calculating more would raise the quality in small steps compared to the time spent.

### Quality estimation by pathway and Gene Ontology over-representation

Hierarchical clustering of gene expression microarray data is often followed by a study to find subtrees with over-representation of genes annotated to some specific Gene Ontology term or pathway [22]. Such over-representation gives information about which biological processes have been active during the experiments as well as suggests biological function for the genes lacking annotations. It would be desirable for the approximate hierarchical clustering to reveal mostly the same pathways and Gene Ontology terms. In order to discover over-represented terms, we used the web-based toolset g:Profiler [23] and studied how many of the highly over-represented terms (p-value below 10^-10^) come up over-represented in the approximate clustering. We defined the quality measure GO50, representing the percentage of highly over-represented terms for which the negative logarithm of the p-value drops at most by 50 percent (*e.g. *from *p *= 10^-12 ^to *p *= 10^-6^). Similarly, GO25 marks the percentage of terms with maximum drop by 25 percent in the negative logarithm of the p-value (*e.g. *from *p *= 10^-12 ^to *p *= 10^-9^).

We have carried out experiments for the Shyamsundar05 dataset for the same set of parameters as in the experiments with JDR quality measure. The results for 20 pivots are presented in Figure 4BC, results for 5 and 10 pivots in Additional file 1. The analysis of full clustering dendrogram revealed 338 highly over-represented Gene Ontology terms and pathways in the subtrees (*p *< 10^-10^). Figure 4B shows how large percentage of these 338 preserved at least 50% of the initial p-value (GO50), Figure 4C presents the same for the 25% limit (GO25).

As with JDR, it is obvious that the two measures rise with the number of calculated distances, reaching close to 100% in the right edge of figures. It is also beneficial to mix heuristics based and random distances, with *s *= 0.5 being fairly good in practice. With more pivots, higher quality can be achieved with the same number of distances. To conclude, these measurements suggest using *q *= 20 pivots and *s *= 0.5 proportion of similarity heuristics based distances.

**Figure 6:**
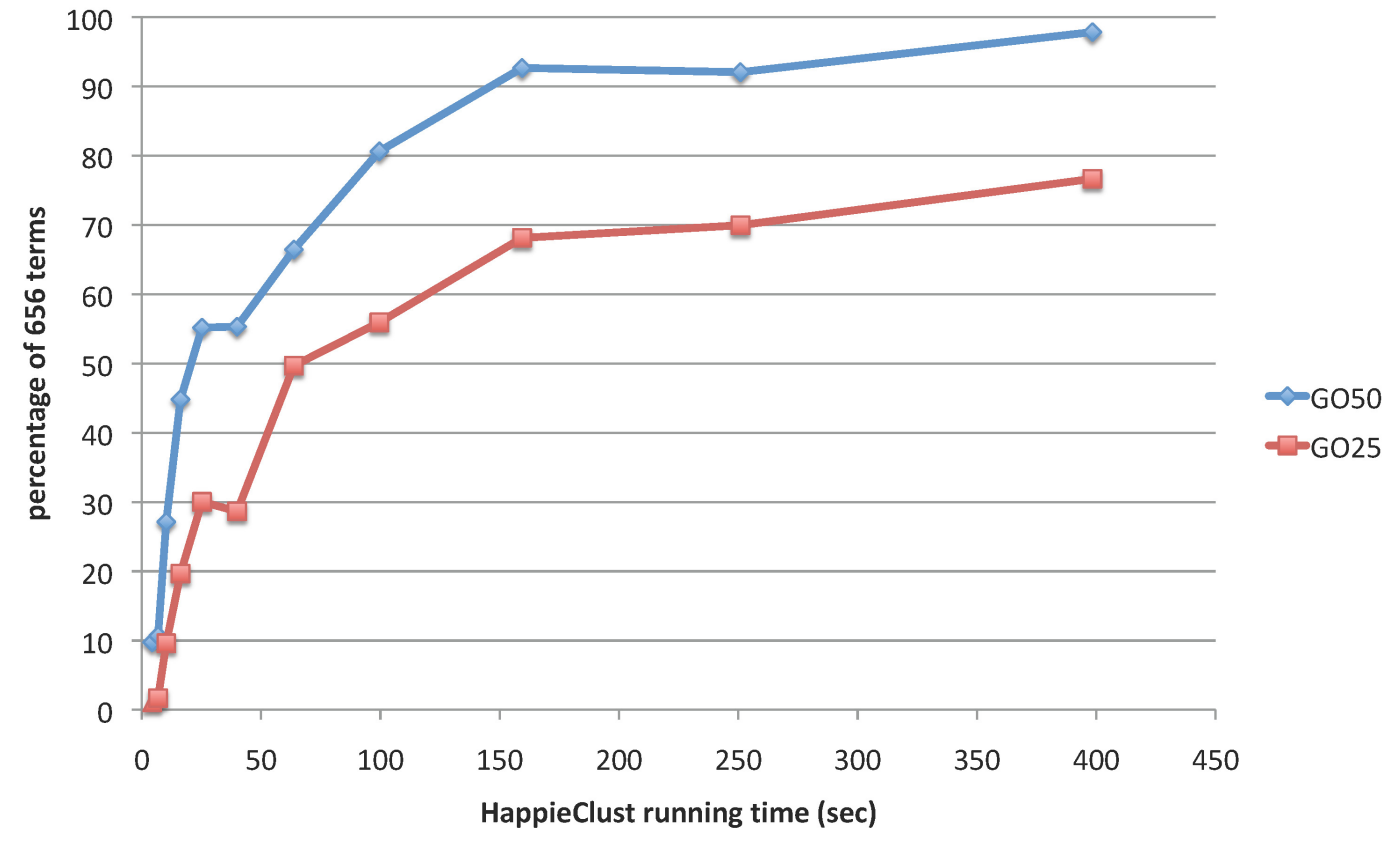
**GO50 and GO25 quality for different running times of HappieClust**. Gene Ontology and pathways annotations based quality for data=Lukk08. GO50 and GO25 quality for data=Lukk08 with different running times of HappieClust, whereas the running time of full hierarchical clustering was 9695 seconds.

**Figure 7:**
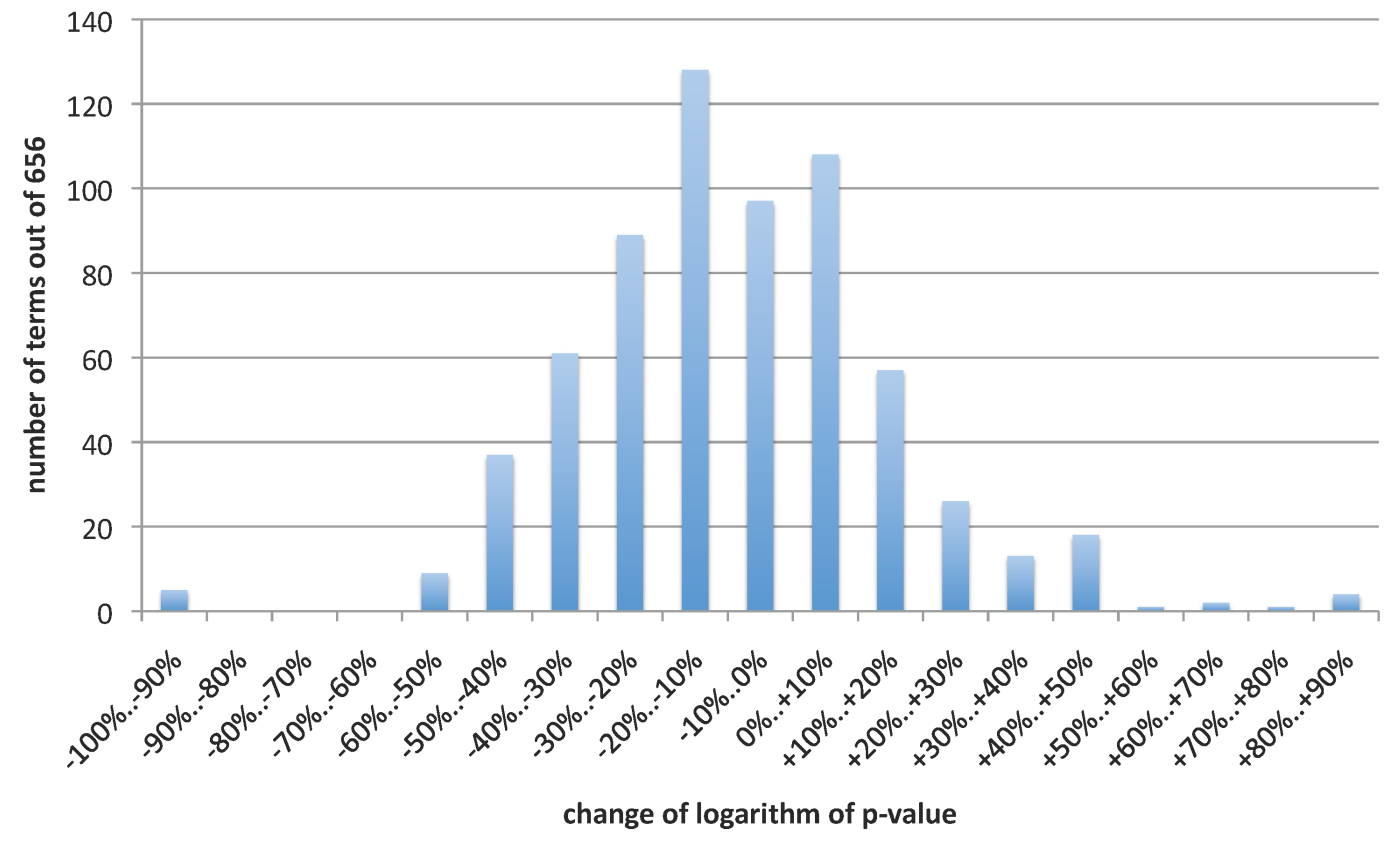
**Gene Ontology based comparison of a run of HappieClust and full AHC**. Distribution of changes in p-values from full clustering to HappieClust with *s *= 0.5, *q *= 20, *m *= 10^7^. HappieClust running time was 400 seconds, whereas full hierarchical clustering spent 9695 seconds.

Finally, we performed the Gene Ontology and pathways annotations based quality evaluation on the Lukk08 dataset for *q *= 20 and *s *= 0.5. The analysis of full clustering dendrogram revealed here 656 terms. Figure 6 shows the GO50 and GO25 quality measures of HappieClust results for 10^5 ^to 10^7 ^calculated distances, taking 4 to 400 seconds of running time. Figure 7 shows the distribution of changes in the p-values going from full hierarchical clustering to approximate hierarchical clustering with 10^7 ^distances. The histogram illustrates the facts that only 14 out of 656 terms have dropped more than 50%, 139 have dropped 25–50%, 273 have dropped 0–25%, and 230 have become more significant, with the median of the distribution at -10%. This indicates that we have found most of the biologically meaningful clusters more than an order of magnitude faster (9695 seconds for full and 400 seconds for approximate clustering).

### Quality estimation by subtree content conservation analysis

As the third measure of quality, we investigated how well each subtree *T *of the full AHC dendrogram was conserved in the approximate dendrogram *D'*. The structure of the subtree was dropped and it was considered as a set of objects. Conservation was measured using the following formula:

$$conservation(T,D^{\prime })=\underset{T^{\prime }\text{ is a subtree of }D^{\prime }}{\max}\frac{\left|T\cap T^{\prime }\right|}{\max(|T|,|T^{\prime }|)}.$$

For instance, a conservation value of 0.7 can be interpreted as at least 70 percent of genes in *T *are part of some subtree *T' *in *D' *such that they form at least 70 percent of *T'*. Such percentages are important when working with gene lists obtained from the subtrees in a dendrogram. In order to visualize the result for all subtrees, the conservation values over similar subtree sizes were averaged.

**Figure 8:**
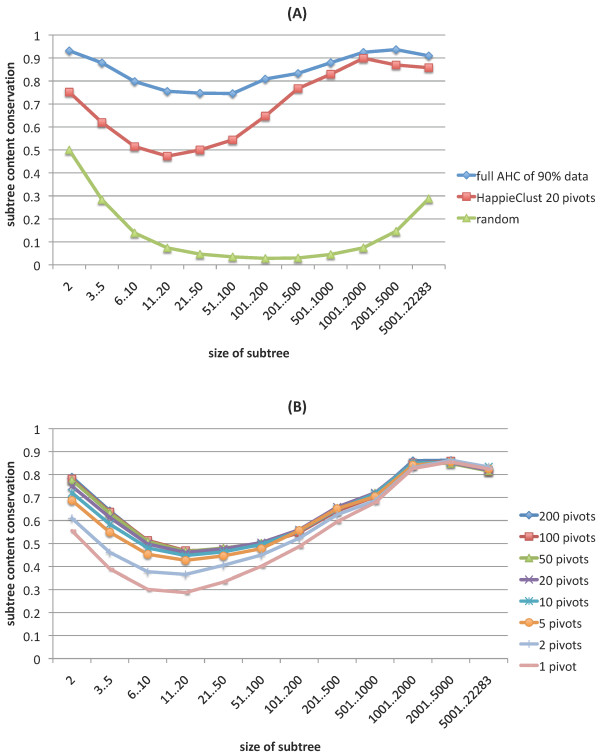
**Subtree content conservation analysis**. Subtree content conservation values for the HappieClust dendrogram of the Lukk08 dataset using *q *= 20, *s *= 0.5 and *m *= 10^7 ^are contrasted with (A) the same values for a random dendrogram and for the full AHC dendrogram of the Lukk08 dataset with 10 percent of conditions removed; (B) the same values for different numbers of pivots.

We calculated the conservation values for the HappieClust dendrogram of the Lukk08 dataset using *q *= 20, *s *= 0.5, *m *= 10^7^, see Figure 8A. As a comparison, we plotted the conservation values for a random dendrogram, obtained by joining randomly chosen clusters at each step. Also, we took the Lukk08 dataset, removed randomly chosen 10 percent of condition columns, and performed full AHC. A similar reduction of gene expression profiles was used in [24]. The difference between full AHC applied to the full and partial datasets shows the kind of variance we can expect when 10 percent of the data is missing. Figure 8A shows that for the clustering of partial data, the conservation value varied from 0.75 to 0.95, and for HappieClust with *m *= 10^7 ^it varied from 0.5 to 0.9. For large clusters with more than 200 elements, HappieClust performed almost as well as full AHC on the partial data. This indicates that the approximation error of HappieClust is almost as small as the natural variance in the data.

We also tested different numbers of pivots with a fixed number of calculated distances. Results in Figure 8B show that the quality improved while moving from 1 pivot to 10 or 20 pivots but then it levelled off. As the calculations take longer for more pivots, it once more confirms that *q *= 20 is a good choice.

As an example we took the subtree *T *of the full AHC dendrogram which had the highest over-representation of some narrow Gene Ontology term (with less than 300 gene annotations). This term turned out to be muscle contraction (GO:0006936). The subtree *T *and its best matching approximation *T' *had 593 and 545 probes corresponding to 496 and 478 genes, respectively. As the overlap of *T *and *T' *was 383 probes, the conservation score was 383/593 ≈ 0.65. Note that this is slightly below 0.8 which is the average conservation for subtrees of size 501..1000 (Figure 8A). These subtrees *T *and *T' *contained 64 and 63 genes out of all 172 genes annotated to muscle contraction, with p-values 2.22·10^-64 ^and 1.89·10^-62^, respectively. The overlap of these 64 and 63 genes was 59, *i.e.*, almost all of the muscle contraction related genes were the same in these subtrees of full and approximate dendrograms, despite the lower conservation score.

## Discussion

Our experiments show that HappieClust achieves very similar results to the full hierarchical clustering more than an order of magnitude faster. In the following, we discuss some ideas that may further improve HappieClust.

Pivots are currently chosen randomly. However, there are two things one could try to avoid. First, if two pivots are similar to each other, then they filter out about the same subset of pairs. Therefore, one might try to choose the pivots to be pairwise non-similar. Second, if a pivot is an outlier in the dataset, then all the objects in the dataset might be of similar distance from it and the pivot would not work as a filter. This encourages to experiment with pivot choice strategies in the future.

Another point of possible advancement is the choice of distances. Once a sub-cluster is formed in the hierarchical clustering process, the distances between the objects inside the cluster do not matter anymore. This suggests a different strategy for the whole workflow. Instead of calculating all the distances at once, the algorithm might interleavingly calculate distances and perform merging steps. The technique of pivots could also be potentially used more than once in the process.

## Conclusion

Agglomerative hierarchical clustering is a technique often used in the analysis of large high-dimensional datasets. Current agglomerative hierarchical clustering algorithms depend on the calculation of all pairwise distances in the dataset. For many possible applications this process is too slow as the number of distances is quadratic in the number of objects to be clustered.

This inspired us to develop a new approach, approximate hierarchical clustering, for which we have implemented a fast algorithm HappieClust. According to our experiments, it achieves very similar results to the full hierarchical clustering more than an order of magnitude faster. HappieClust makes use of similarity heuristics to quickly find many pairs of similar data objects, without calculating all pairwise distances. The heuristics are based on pivots, a technique which is often used in the similarity search community [14]. The technique could possibly be used also for other clustering methods and data analysis apart from agglomerative hierarchical clustering.

The running time of HappieClust can be easily controlled and it achieves a better approximation when given more time. This is useful in interactive and web-based applications where users expect fast response and unknown running time is undesirable. The majority of datasets can be clustered with HappieClust on personal computers, as the minimal required amount of main memory is the size of the initial dataset. We measured the quality of HappieClust approximation using three methods. The first, joining distance ratio, showed that approximation gets close to minimizing the same function that is greedily minimized by the full clustering algorithm. The second applied to clustering of gene expression data and studied the over-representation of gene ontology terms and pathways in the subtrees of the dendrogram. It showed that almost all highly over-represented terms in the full hierarchical clustering dendrogram are still over-represented in the approximate dendrogram, whereas the p-values do not lose strength too much. This indicates that biologically relevant clusters are formed and biological interest can still be evaluated. The third measure studied the subtree content conservation. It pointed out that the subtrees of approximate and full AHC dendrograms are similar, and thus, the corresponding gene lists have high overlap.

HappieClust is intuitive and truly unsupervised. The only new parameter compared to full hierarchical clustering is *m*, the number of distances to be calculated. Higher values of *m *result in better approximations of full hierarchical clustering. As the running time of HappieClust is linear in *m*, it is possible to choose *m *according to the given time constraints. If needed, more elaborate analysis techniques can be used upon gaining the first insights from approximate hierarchical clustering.

## Competing interests

The authors declare that they have no competing interests.

## Authors' contributions

MK developed and implemented the HappieClust algorithm, carried out the computational experiments and wrote the draft of the article. JV directed the project and was involved in revising the manuscript. Both authors read and approved the final manuscript.

## Supplementary Material

Additional file 1 The influence of parameters on approximation quality. Figures illustrating the influence of the number of pivots on the JDR, GO50 and GO25 quality measures. Click here for file
