# Supplementary material for: Fast approximate hierarchical clustering using similarity heuristics
Source: BioData Min. 2008 Sep 22;1:9. doi: 10.1186/1756-0381-1-9 (PMC2561018; doi:10.1186/1756-0381-1-9)
Supplement: Additional file 1 — The influence of parameters on approximation quality. Figures illustrating the influence of the number of pivots on the JDR, GO50 and GO25 quality measures. [file 1756-0381-1-9-S1.pdf]

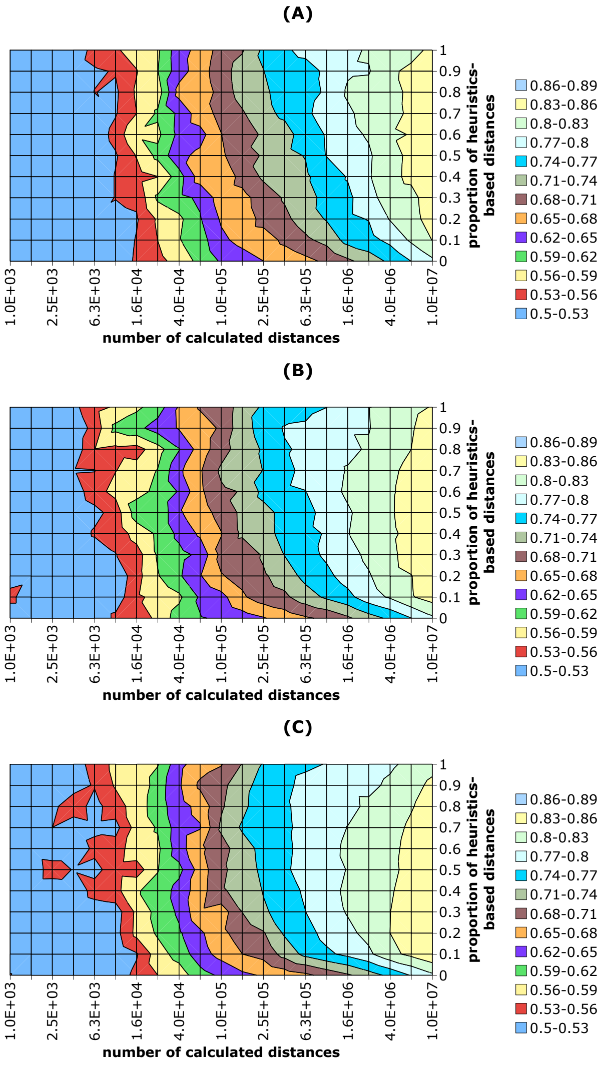

Supplementary Fig. 1: HappieClust JDR quality for data=Shyamsundar05 and different numbers of pivots: (A) $q = 5$; (B) $q = 10$; (C) $q = 20$.

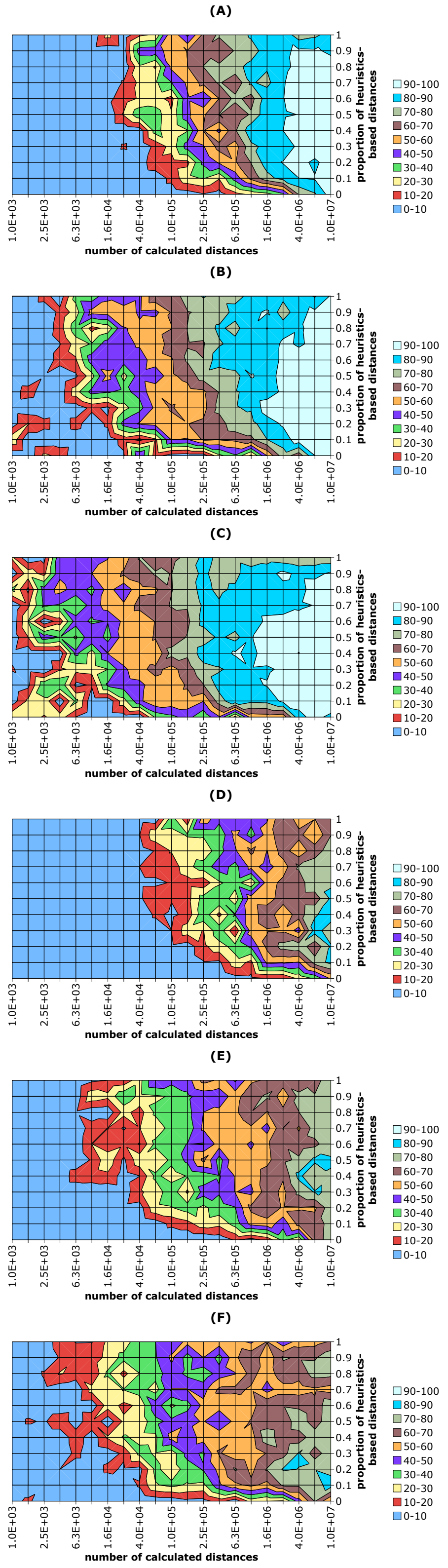

Supplementary Fig. 2: GO50 and GO25 quality of HappieClust for data=Shyamsundar05 and different numbers of pivots. (A) $q = 5$, GO50; (B) $q = 10$, GO50; (C) $q = 20$, GO50; (D) $q = 5$, GO25; (E) $q = 10$, GO25; (F) $q = 20$, GO25.
